# Resolving microbial membership using Abundance and Variability In Taxonomy (*‘AVIT* )

**DOI:** 10.1038/srep31655

**Published:** 2016-08-17

**Authors:** Anirikh Chakrabarti, Jay Siddharth, Christian L. Lauber, Mathieu Membrez, Bertrand Betrisey, Carole Loyer, Chieh Jason Chou, Zoltan Pataky, Alain Golay, Scott J. Parkinson

**Affiliations:** 1 Nestlé Institute of Health Sciences, Lausanne, Switzerland; 2 Service of Therapeutic Education for Chronic Diseases, WHO Collaborating Centre, University Hospitals of Geneva and University of Geneva, Geneva, Switzerland

## Abstract

Development of NGS has revolutionized the analysis in microbial ecology contributing to our deeper understanding of microbiota in health and disease. However, the *quality*, *quantity* and confidence of summarized taxonomic abundances are in need of further scrutiny due to sample dependent and independent effects. In this article we introduce **‘AVIT** (*Abundance and Variability In Taxonomy*), an unbiased method to enrich for assigned members of microbial communities. As opposed to using *a priori* thresholds**, ‘AVIT** uses inherent abundance and variability of taxa in a dataset to determine the inclusion or rejection of each taxa for further downstream analysis. Using *in-vitro* and *in-vivo* studies, we benchmarked performance and parameterized **‘AVIT** to establish a framework for investigating the dynamic range of microbial community membership in clinically relevant scenarios.

NGS has greatly expanded our understanding and knowledge of microbes and their importance in a variety of habitats, by allowing for sequencing an ever-increasing *number of samples*^1^ and *depth*^2^. Simultaneously, recent work has reiterated the importance of microbes to human health^3^,^4^,^5^,^6^,^7^,^8^,^9^,^10^. However, there exists disconnects between advancements in sequencing capability and our understanding of community membership. Additionally, our understanding is confounded by uncertainties in technical and bioinformatic assignment of taxonomy to ribosomal sequences. These limitations handicap our ability to evolve towards an understanding of integrated community metabolism and dynamics.

NGS data processing in microbial ecology (ensuring balance between “*quantity*” and “*quality*”) remains an important area for development. Current strategies for the removal of spurious data encompass platform specific methods, pre-processing assessment of quality and post processing removal of data-points using *a priori* thresholds. For example, flowgram de-noising is specific to 454 Life Sciences Pyrosequencer and does not apply to other sequencing NGS methods^11^,^12^. In case of Illumina, CASAVA filters sequences on a per-read basis. Additionally, Caporaso *et al.* have suggested strategies to avoid exaggerated diversity estimates using Illumina reads^13^. Bokulich *et al.* demonstrated how high-quality read length and abundance were the primary factors differentiating authentic and spurious reads produced by different sequencing methods^14^. They presented guidelines for user-defined quality-filtering enabling efficient extraction of high-quality data and facilitating interpretation of Illumina sequencing reads. These methods focus on platform-specific solutions but stop short of addressing other mechanisms contributing to spurious data inclusion. The community could benefit from a consistent methodology addressing these concerns in a platform independent manner. Current data-processing methods cull low abundance OTUs or model the abundance and distribution of all OTUs in a data set, to reduce variability and increase confidence in statistical measures of community composition^15^. *A priori* cutoffs risk discarding low abundant but omnipresent members that contribute to the metabolic potential of the community.

This concept led us to explore the utility of concurrently considering both taxon variability and abundance in analysis of complex microbial communities using NGS platforms. We introduce **‘AVIT** (*Abundance and Variability In Taxonomy*), an unbiased method to use the available metrics (relative abundances and variability in the whole study) in unison to remove potentially erroneous members while retaining low-abundant legitimate members of the microbial community. Using defined *in-vitro* and *in-vivo* studies with *a priori* knowledge of sample composition, we identified thresholds at which high quality noise reduction was achieved and benchmarked **‘AVIT** performance. Key advantages of **‘AVIT** include: a) removal of spurious members, b) inclusion of members known to be present across 10^6^ abundance range (otherwise rejected by *a priori* abundance cutoffs), and c) optimizing the balance of membership and accounted variability in downstream analysis. By applying **‘AVIT** to a clinical study^16^, we identified the spectrum of microbial fecal community membership from clinical samples. We provide a classification based on resolution to assign a confidence regarding accurate community membership, which will facilitate downstream analysis and hypothesis generation. Overall, the **‘AVIT** framework provides inferences that cannot be achieved using preexisting approaches, and its broad application could enhance our understanding of microbial communities.

## Material and Methods

### Abundance and Variability In Taxonomy (‘AVIT) Algorithm

**‘AVIT** algorithm uses the outputs from datasets in the form of taxa counts obtained after pre-processing, OTU and taxonomy assignment steps common in many 16S pipelines. **‘AVIT** filters out elements by taking into account taxon abundance and variability in a sample and across samples to identify members to be analyzed. Abundance of a member is quantified by the raw count observed post sequencing. While variability can be quantified in different ways, we specifically are referring to either the sample mean (average) or study mean (average) as a measure of variability.

**Figure 1:**
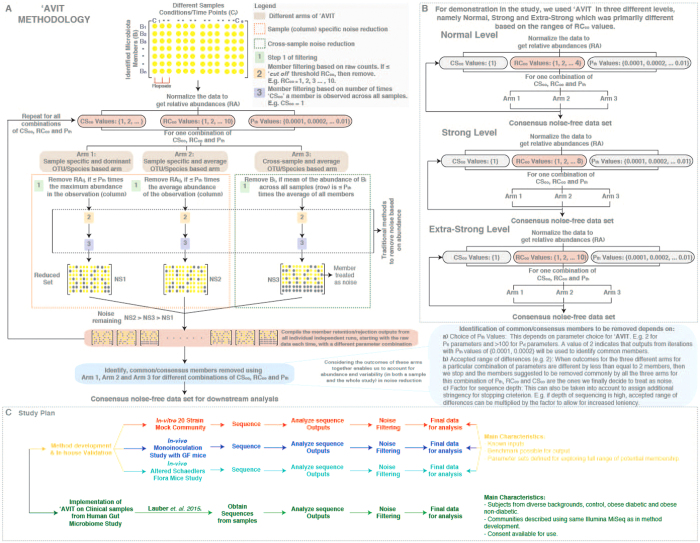
Abundance and Variability In Taxonomy (***‘AVIT***) methodology. (**A**) In ‘***AVIT*** we look into both the column (individual sample) and row (across samples) and using metrics like average abundance within a sample, maximum abundance within a sample and average abundance over the whole data-set, we take into account not just abundance but also variability into removal of noise (potentially erroneous species) from 16S taxonomic data. Individual characteristics of each arm can be seen in Supplementary materials S1-S2. (**B**) Different levels/modes of ‘***AVIT*** used in the current study; normal, strong and extra-strong and its corresponding parameters. Primarily, the three levels used for demonstration in the current study differed in the ranges of parameters for the *raw count cut-off* (**RC**_**co**_). (**C**) Current study plan. We used a combination of *in-vitro* (20 strains mock community) and *in-vivo* (monoinoculated GF mice and Altered Schaedlers Flora mice) to develop, identify parameters and validate ‘***AVIT*** methodology in house. Subsequently, we applied ‘***AVIT*** to clinical samples from Lauber *et al.*^16^ study (including lean healthy, obese diabetic and obese non-diabetic patients) to assess the applicability of the methodology and highlight the implications of our findings.

Implementation of **‘AVIT** was carried out using MATLAB (The Mathworks, Natick, MA) on an Apple workstation (Apple, Cupertino, CA) and involved the following steps (Fig. 1):

1. The initial step of **‘AVIT** algorithm normalizes the raw counts of each identified member (***B***_***i***_, where ***i*** is a member of the ***n*** originally identified members being either OTUs or taxa counts) within each sample (***C***_***j***_, where ***j*** corresponds to one sample amongst the ***s*** samples) to the sum of all the members identified in the corresponding sample obtaining the *relative abundances* (***RA***_***ij***_).
2. This is followed by a core filtering sequence within **‘AVIT**, based on a combination and ultimately consensus set, derived from choice and application of three parameters: i) *proportionality threshold* (***P***_***th***_), ii) *raw count cut-off* (***RC***_***co***_), and iii) *cross-sample cut-off* (***CS***_***co***_). **P**_**th**_ values are in the form of 0.0001, 0.0002… 0.01. These parameters indicate a threshold allowing for selection/rejection of a measurement as compared to the rest of the dataset. For example, if a relative abundance of a member is less than the chosen **P**_**th**_ multiplied to the maximum relative abundance in a sample, then the scheme would reject the member under consideration. **RC**_**co**_values are integers (eg. 1, 2… 10) indicating a threshold to allow for selection or rejection of a member based on the raw count observed. For example, using an **RC**_**co**_ of 2 would filter out all measurements with raw counts ≤2. **CS**_**co**_values are also integers indicating the threshold used to accept or reject a member based on how many times it is observed across different samples. For example, using a **CS**_**co**_of 2 would result in filtering out a member observed only twice across all samples in the study. Using a combination of these parameters, we take into account sample specific and cross-sample information to define retained members. Based on a combination of choices of the parameters discussed earlier (e.g. **P**_**th**_ = 0.0001, **RC**_**co**_ = 2, **CS**_**co**_ = 1), we perform three parallel steps to filter the original dataset as outlined below:

**Arm 1 Sample specific and dominant member level based arm –** using the dataset in the form of relative abundances (***RA***_***ij***_), the algorithm filters out the ***RA***_***ij***_ values in any particular column (corresponding to a sample) which are ≤**P**_**th**_ times the maximum ***RA***_***ij***_ for the corresponding column. Consequently, based on the original raw counts, it will remove the corresponding entries in the dataset that are ≤**RC**_**co**_. Finally in the reduced/filtered dataset, it would check if any member was observed in different samples greater than the initially chosen **CS**_**co.**_If not, then it would filter out the corresponding member. This finally results in a reduced/filtered subset of the initial dataset for the corresponding parameters deployed for the filtration process.

**Arm 2 Sample specific and average member level based arm –** Based on the relative abundances (***RA***_***ij***_), in this arm, the algorithm would filter out the ***RA***_***ij***_ values in any particular column (corresponding to a sample) which are ≤**P**_**th**_ times the **mean (average)** ***RA***_***ij***_ for the corresponding column. Consequently, it would filter further based on the original raw counts based on **RC**_**co**_ and presence across different samples based on **CS**_**co**_as discussed earlier. This results in a reduced/filtered subset of the initial dataset for the corresponding parameters that was used to perform the cutoff.

**Arm 3 Cross-sample and average member level based arm –** Using relative abundances (***RA***_***ij***_) dataset, the algorithm would first calculate the **mean** of the **RA**’s (**m*****RA***_***i***_) for each of the identified members. It would then filter out the members for which the **m*****RA*** are ≤**P**_**th**_ times the mean (average) **m*****RA***_***i***_ across all the identified members. Consequently, it would filter further based on the original raw counts based on **RC**_**co**_ and presence across different samples based on **CS**_**co**_as elaborated earlier. Finally, it results in further reduced/filtered subset of the initial dataset for the corresponding parameters used for data filtration.

Using these three arms (1, 2 and 3) and the corresponding starting filtering parameters (i.e. **P**_**th**, _**RC**_**co**_and **CS**_**co**_), the proposed method would have three parallel arms to take into account only the abundances within each sample (arm 1), both abundance and variability within each sample (arm 2) and both abundance and variability within the whole study (arm 3) and obtain different possible filtered states of the initial dataset which was the original input data. An iteration of step 2 is performed with all other possible combinations of parameters, within the initial starting range; to obtain all the possible filtered datasets. Each iteration refers to an individual independent run, starting with the raw data each time, with a different parameter combination of **P**_**th**, _**RC**_**co**_and **CS**_**co**_.

The final step is to collect and compile all the information contained within the different filtered datasets obtained after the iterations in step 2. This allows us to simultaneously account for abundance and variability within/across all samples. Upon completion of all individual runs, we identify the common members that were removed using the three arms and across different ranges of **P**_**th**, _**RC**_**co**_and **CS**_**co**_parameters. Consensus is primarily an outcome of three components. First based on whether you are using **P**_**6**_ or **P**_**d**_ parameters (corresponding to diverse community with members varying over six order of magnitude ranges or dominant members only community) for the **‘AVIT** implementation, outcomes of selected **P**_**th**_ values are only taken into consideration. For example, for **‘AVIT** implementation with **P**_**6**_parameters, the outcomes corresponding to the first two **P**_**th**_ values of 0.0001 and 0.0002 are taken into account (along with combinations with other parameters **RC**_**co**_ and **CS**_**co**_). Similarly for **‘AVIT** implementation with **P**_**d**_parameters, the outcomes for the first 100 or more **P**_**th**_ values are taken into account. With all these different outcomes, i.e. with different combinations of **P**_**th**_ (based on the parameter choice as discussed above), **RC**_**co**_ and **CS**_**co**_, we identify the lowest combination of **P**_**th**_, **RC**_**co**_ and **CS**_**co**_, such that their outputs (marked by members removed and retained) are similar within an accepted range (stopping criterion). This accepted range of variability is user defined. For example, in the current study, we used a value of 2. This indicates that when the outcomes for the three different arms for a particular combination of parameters (**P**_**th**_, **RC**_**co**_ and **CS**_**co**_) are different by less than equal to 2 members, then we stop and the members suggested to be removed commonly by all the three arms for this lowest combination of **P**_**th**_, **RC**_**co**_ and **CS**_**co**_ are the ones we finally decide to treat as noise. An additional control over this accepted range of variability can de obtained by using a factor for the depth of sequencing. For example, if you have deep sequencing, you can relax the accepted range of variability by a factor.

For example, in the current study, we employed three different stringency levels of noise reduction using **‘AVIT** for demonstration purposes, namely normal, strong and extra-strong (Fig. 1B).

- Normal noise reduction was characterized by consensus filtering of members based on parameter choices of **P**_**th**_ = 0.0001, 0.0002… 0.01, **RC**_**co**_ = 1, 2… 4 and **CS**_**co**_ = 1.
- Similarly, strong noise reduction was characterized by consensus filtering of members based on parameter choices of **P**_**th**_ = 0.0001, 0.0002… 0.01, **RC**_**co**_ = 5, 6… 8 and **CS**_**co**_ = 1.
- And finally, extra-strong noise reduction was characterized by consensus filtering of members based on parameter choices of **P**_**th**_ = 0.0001, 0.0002… 0.01, **RC**_**co**_ = 9, 10 and **CS**_**co**_ = 1.

Therefore, by employing different ranges of parameter values for **P**_**th**, _**RC**_**co**_and **CS**_**co**_ and thereby accounting for sample specific and study specific abundances and variabilities, the method was able to obtain different yet reliable filtering of erroneous members.

### *In-vitro* mock community composition and 16S rRNA gene analysis

**Figure 2:**
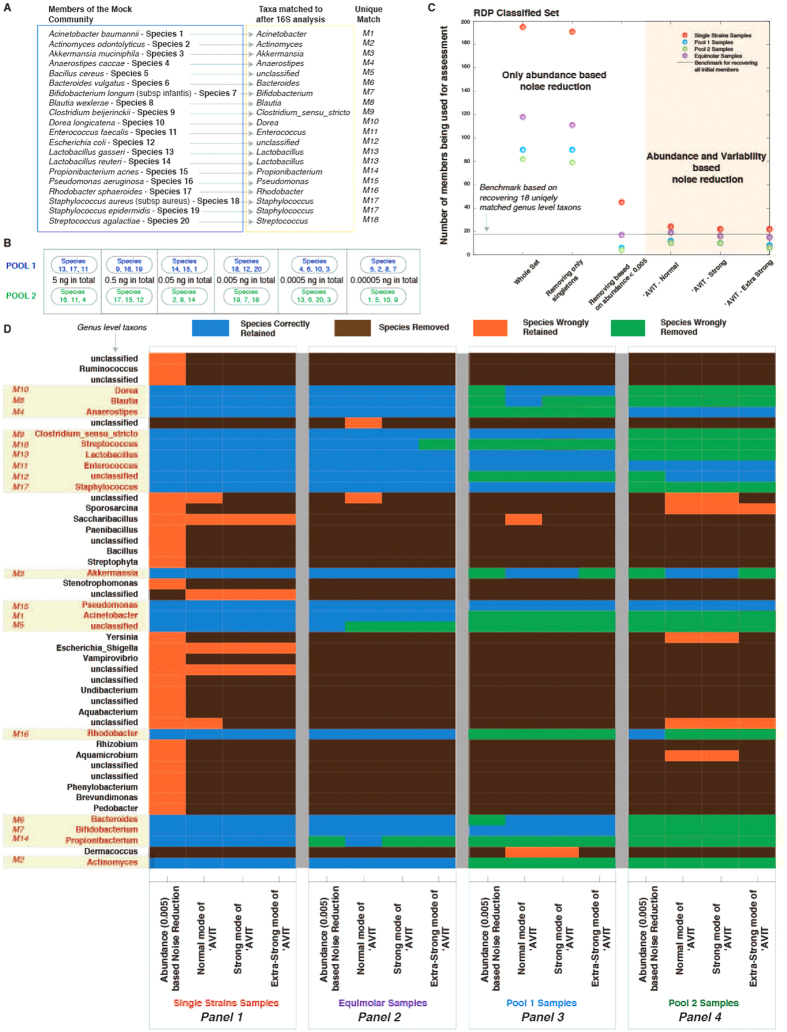
Application of ‘***AVIT*** at different levels normal, strong and extra strong, on separate *in-vitro* Equimolar, Single Strain and staggered pool samples of 20 strain mock community. (**A**) Strains used in the study and corresponding matches at the genus level after using the 16S pipeline. (**B**) Composition of the staggered pools with the corresponding species and their concentrations. (**C**) Number of species retained in different samples upon using abundance only noise reduction and using ‘***AVIT*** at different stringency levels. (**D**) Species correctly/incorrectly retained or removed in 0.005 relative abundance based cutoff and different levels of ‘***AVIT*** application to single strain, equimolar and staggered pool samples.

Genomic DNA from 20 individual bacterial species (Fig. 2A, Supplementary Table S1) was obtained from DSMZ to compose a mock community. V4 variable region of 16S rRNA genes were amplified using a unique barcoded primer with the PCR conditions described in Caporaso *et al.*^17^. Amplicons were then quantified using PicoGreen and combined in equal molar amounts to form a single pool for paired-end sequencing on the MiSeq platform (Illumina Inc, San Diego CA, USA), using version 2.0 chemistry.

The mock community was used in two parts; a) First, we took a mixture of 40 *in-vitro* samples, including 20 single strain samples, 6 equimolar pool samples and 14 staggered pools with 6 orders of magnitude different concentrations of the members (details in Supplementary data *Stagger_Proportions_For_InVitro_Pools.xls*), b) Second, we created four *in-vitro* sets for analysis; Equimolar set (*EMS*), Pool 1 set (*P1S*), Pool 2 set (*P2S*) and Single Strain set (*SSS*). In case of *EMS*, 20 strains were mixed in equimolar ratios (10 ng/μL in total) and 15 replicates were sequenced across three MiSeq sequencing machines. In *P1S* and *P2S*, we varied the abundance of 20 species (in different orders) over six orders of magnitude (5 ng/μL to 0.00005 ng/μL) as illustrated in Fig. 2B and ran 17 replicates across three MiSeq machines.

The demultiplexed, fastq formatted sequences resulting from MiSeq runs were analysed using MOTHUR version 1.29.1 following the MiSeq SOP (dated 14^th^ Nov 2014)^18^. The paired end reads were combined to form a contig for each amplicon, which were then trimmed to homogenous length. Sequences with homopolymers and low quality scores were also removed from the data. This was then used to perform an alignment using the SILVA release 119, trimmed to the V4 region. The alignment was then trimmed followed by chimera removal using UCHIME^19^, the sequences were then classified using Mothur against GreenGenes (GG) (release 13_5)^20^, RDP (release 9) and SILVA database (release 119) using the wang method for classification with 1000 iterations (http://www.mothur.org/wiki/MiSeq_SOP). The resultant tables were then used as input for further analysis in the **‘AVIT** strategy.

### *In-vivo* experiments

Procedures were approved by “Office Vétérinaire Cantonal du canton de Vaud” Lausanne, Switzerland (Authorization number 2718). All procedures were carried out in accordance with the approved guidelines. All germfree male C57BL/6J mice were purchased at 8 weeks of age from Charles River Laboratories (L’Arbresle, France). Upon arrival, mice were housed individually under a 12 h light/dark cycle for 1 week. All mice (n = 15) were given autoclaved water and γ-irradiated (40 kGy) chow diet (R03-40, Safe diets, Augy, France). Fecal samples from all mice (n = 15) were obtained 1 day before the intervention (day -1). On day 0, 7 mice were randomly selected and treated with 10^8^ CFU/mL *E. coli* in drinking water for 14 days and their fecal samples were collected at 1 day after and 14 days after the treatment. The remaining 8 mice were kept in a germfree condition and their fecal samples were collected at day 14. Altered Schaedler Flora (ASF) mice fecal samples were provided by Nicola Harris and Kathy McCoy.

### Clinical Study

Details about the study population, study design, intervention, fecal sample collection, DNA extraction, PCR, sequencing and analysis are provided in Lauber *et al.*^16^ (also provided in Supplementary materials).

### Accession Numbers

The data reported in the paper will be provided on SRA database (SRP079895). This data includes the raw sequences for both the *in-vitro* and *in-vivo* studies. The raw data for the clinical fecal samples is associated with the manuscript by Lauber *et al.*^16^.

### Supplemental Information

Supplemental information includes supplementary text explaining the inner workings, different implementation states and parameterizations of **‘AVIT**. Additionally it also includes additional results on the different studies using different parameter sets. Overall, it includes supplemental text, eight supplemental figures, one supplemental table, twelve excel sheets with data and one zip file with the code (in MATLAB) and demonstration of **‘AVIT**.

## Results

In this study we introduce **‘AVIT** - an approach considering both abundance and variability within and across samples to define members of a microbial community (Fig. 1). Using three key parameters: i) *proportionality threshold* (***P***_***th***_), ii) *raw count cut-off* (***RC***_***co***_), and iii) *cross-sample cut-off* (***CS***_***co***_), split over three parallel arms, we take into account the abundances within each sample (*arm 1*), both abundance and variability within each sample (*arm 2*) and both abundance and variability within the whole study (*arm 3*) (materials and methods). Additionally, **‘AVIT** can be implemented in different stringency levels (normal/strong/extra-strong) using different ***RC***_***co***_ and for different communities using different combinations of ***P***_***th***_ and ***RC***_***co***_. We used a combination of *in-vitro*, *in-vivo* and clinical studies for development, parameterization and benchmarking of **‘AVIT**. *A priori* knowledge of the sample composition facilitated identification of threshold values (parameters) to improve noise reduction. However, defining such parameters for clinical samples of unknown composition is more challenging. Therefore, using the benchmarked parameters coupled with different stringency levels, we explored the spectrum of potential membership of microbes in clinical samples.

### *In-vitro* mock community and ‘AVIT

Many factors (sample composition, type, source, and sample independent factors like DNA extraction, PCR, reagents, laboratory error and sequencing machines) contribute to the variability observed in any study^21^,^22^. Knowing this, we began with an *in-vitro* mock community of 20 defined bacterial taxa (Fig. 2A, methods) and applied **‘AVIT** to filter noise and attempted to identify only known members of the mock community.

First, we took a mixture of 40 *in-vitro* samples including single strain, equimolar and staggered pools and analyzed them using a single MiSeq run (methods). We obtained 132–148 genus level taxa after classifying the sequences. Removing singletons (members identified in only one sample), reduced ∼7.2% noise in RDP, 2.34% in SILVA, and 1.90% in GG classified datasets (Supplementary materials S1-S2). Using a relative abundance based cutoff of 0.005 (*Rabc0.005*), we reduced 92.8% noise in RDP, 92.18% in SILVA, and 90.65% in GG classified datasets. In comparison, using **‘AVIT** at different levels (normal, strong and extra-strong), we obtained ∼93% noise reduction in normal level, ∼95% in strong level and ∼97% in extra-strong level consistently across the RDP, SILVA and GG classified datasets. Effects of the individual arms of **‘AVIT** (corresponding to the differential use of relative abundance and variability) and demonstration of the stringency levels (normal-strong-extra-strong) is provided in Supplementary materials S1-S2.

Next we analyzed differences emerging from sample independent sources, e.g. different machines. We analyzed four *in-vitro* sets; Equimolar set (*EMS*), Pool 1 set (*P1S*), Pool 2 set (*P2S*) and Single Strain set (*SSS*) (methods, Fig. 2B). These sets were selected as defined surrogates for mimicking biological samples. We obtained 118 genera in *EMS*, 90 in *P1S*, 82 in *P2S*, and 195 in *SSS* (taxonomic assignments tabulated in Supplementary data). Removing singletons had a negligible effect as we still retained 111 members in *EMS*, 90 in *P1S*, 79 in *P2S* and 191 in *SSS* (Fig. 2C). Using *Rabc0.005*, we retained 17 members in *EMS*, 6 in *P1S*, 4 in *P2S* and 45 in *SSS*. Using **‘AVIT**, we retained 19/16/15 (normal/strong/extra-strong) members in *EMS*. Similarly, we retained 12/10/8 (normal/strong/extra-strong) members in *P1S*, 10/10/6 (normal/strong/extra-strong) members in *P2S* and 24/22/22 (normal/strong/extra-strong) members in *SSS* (Fig. 2D).

*In silico* the 20 mock community members match to 18 distinct genera (Fig. 2A). For *SSS*, **‘AVIT** performed better than the abundance based noise reduction, ∼78% noise reduction in normal level and ∼85% in strong and extra-strong levels were achieved as compared to *Rabc0.005* (Fig. 2D panel 1). In all cases of *SSS*, we retained the original 20 members. Thus in samples with dominant members, **‘AVIT** performed better when compared to *a priori* abundance based cut-offs. Conversely, in the case of *EMS*, using *Rabc0.005*, 17 out of 18 distinct genera were retained (Fig. 2D panel 2). In comparison, we retained 19 members using normal level of **‘AVIT**. However, two members were erroneously retained. While we retained *Propionibacterium*, *Bacillus cereus* was removed. Strong and extra-strong level retained 16/18 and 15/18 distinct genera respectively without any additional inclusion of erroneous members. Using the *EMS* dataset and **‘AVIT**, we are probing the limits of balancing the cost of noise removal at the risk of losing true members.

Using *Rabc0.005* in *P1S*, we retained *Bifidobacterium longum*, *Clostridium beijerinckii*, *Enterococcus faecalis*, *Lactobacillus gasseri*, *Lactobacillus reuteri*, *Pseudomonas aeruginosa*, *Staphylococcus aureus* and *Staphylococcus epidermidis* (Fig. 2D panel 3). Of the three most abundant members in the P1S pool (5 ng/μL), *Rhodobacter sphaeroides* was not retained. The three members at the 0.5 ng/μL range were retained along with *Bifidobacterium longum*, which was at 0.00005 ng/μL. While the inputs differed by 6 orders of magnitude, the output (measured abundance levels) correlated poorly with the initial concentration, illustrating biases introduced by laboratory based steps like DNA extraction, PCR, reagents, laboratory error and sequencing rather than *in silico* methods (Supplementary data). Using **‘AVIT**, we retained additional true members like *Bacteroides vulgatus, Dorea longicatena, Blautia wexlerae,* and *Akkermensia muciniphila*, which were spread across different initial concentration ranges (Fig. 2B). However, we retained additional erroneous members, e.g. *Saccharibacillus* (normal level of **‘AVIT**) and *Dermacoccus* (normal and strong level of **‘AVIT**). Despite this cost, resolution of the mock community by **‘AVIT** (normal level) was 67% better as compared to the abundance only method. We defined the parameter sets used by **‘AVIT** to obtain optimized retention of true members across staggered pools (six-orders of magnitude variable microbial community) as P_6_ parameters (Supplementary materials S3). Using *Rabc0.005* in *P2S*, we retained only highly abundant members in the 5 ng/μL and 0.5 ng/μL pools, (*Anaerostipes caccae*, *Enterococcus faecalis*, *Pseudomonas aeruginosa* and *Rhodobacter sphaeroides)* (Fig. 2D panel 4). In comparison, using **‘AVIT**, we also retained *Escherichia coli* and *Akkermensia muciniphila* at the cost of removing *Rhodobacter sphaeroides*. **‘AVIT** (normal level) was ∼50% better at retaining true members as compared to the abundance only method, however at the cost of inclusion of 5 erroneous members.

In summary, in all cases (*SSS, EMS, P1S and P2S*) mock community retention using **‘AVIT** was equivalent or superior to the *a priori* cutoff. In some cases (*P2S* and *EMS*), the improved community membership came at the cost of noise. Additionally, our findings with mock communities demonstrated that abundance did not necessarily translate to community inclusion.

### ‘AVIT and *in-vivo* studies on Germ-free mice, mice inoculated with *E. coli* and Altered Schaedlers Flora mice

Motivated by the outcomes of using **‘AVIT** on *in-vitro* mock community studies, we investigated the efficacy of **‘AVIT** on (a) Germ-free (GF) mice, (b) GF mice monoinoculated with an *E. coli* strain and (c) Altered Schaedlers Flora (ASF) mice (Fig. 1C). This study design allowed us to have known inputs to benchmark the outputs and performance of **‘AVIT** and to identify parameters for **‘AVIT** for analysis of communities with dominant members.

**Figure 3:**
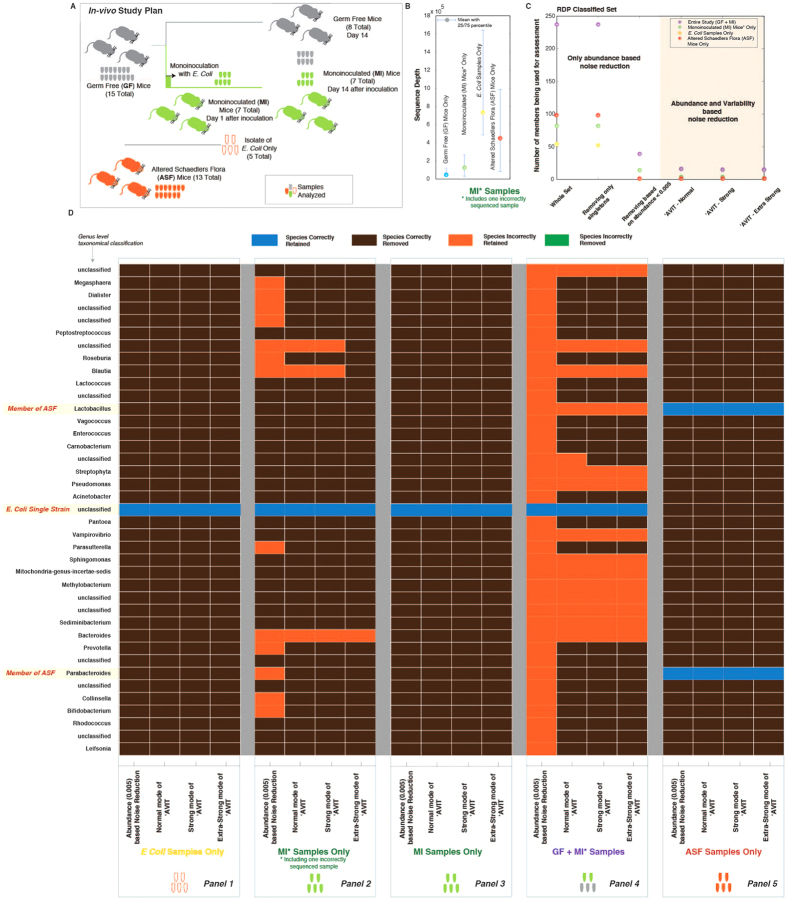
*In-vivo* application of ‘***AVIT*** for a monoinoculation study and analysis of Altered Schaedlers Flora (ASF) Mice fecal samples. (**A**) 15 Germ-free (GF) mice were used for the monoinoculation study. 7 GF mice were monoinoculated with *E. coli* (subsequently refered to as MI mice) and fecal samples were collected on Day 1 and Day 14 after monoinoculation. Additionally fecal samples were collected from all the 15 GF mice before monoinoculation (at Day 0) and from 8 remaining GF mice on Day 14. Additionally, 5 pure *E. coli* samples were used as controls. 13 fecal samples were collected from ASF bred mice. (**B**) Variation of sequence depths observed for different groups of samples. (**C**) Number of members retained after different forms of noise reduction for different samples. (**D**) Members correctly/incorrectly retained or removed in 0.005 relative abundance based cutoff and different levels of ‘***AVIT*** application to the GF + MI, MI* (including one erroneously sequenced sample) samples, MI (excluding the erroneously sequenced sample) samples, *E. coli in-vitro* samples and samples from ASF mice respectively.

For the first part of the study, 7 GF mice were inoculated with *E. coli* (referred further as MI mice) on Day 0 (Fig. 3A). Fecal samples were collected before inoculation (GF), and 1 and 14 days after inoculation. Additionally, fecal samples from the remaining 8 GF mice were collected on Day −1 and Day 14. Additionally, 5 *in-vitro E. coli* culture samples (referred further as EC samples) were used as controls. For all 42 samples, DNA extraction was performed and sequenced using one MiSeq machine. Average sequence depth along with 25 and 75 percentile around the mean for different groups are shown in Fig. 3B.

After classification of the sequences, we obtained 231 genus level taxa in *GF* samples (*GFS*), 82 in *MI* samples (*MIS*), and 54 in *EC* samples (*ECS*) (Supplementary data). Considering all the samples together (whole study together: *WST*) we obtained 237 unique genus level taxa. Removing singletons we retained 237 members in *WST*, 230 in *GFS*, 82 in *MIS* and 52 in *ECS*, therefore having little to no effect of noise reduction (Fig. 3C). Using *Rabc0.005*, we retained 39 members in *WST*, 31 in *GFS*, 14 in *MIS* and 1 in *ECS*.

Output of **‘AVIT** is dependent on the parameters used. Accordingly, parameters identified from the *in-vitro* experiments (P_6_) to optimize retention of true members in a six-orders of magnitude variable microbial community are not applicable. So we identified P_d_, i.e. parameters optimized for community dominated by few dominant members. P_6_ and P_d_ primarily differ based on the ranges of **P**_**th**_ (proportionality thresholds) and **RC**_**co**_ (raw count cutoff), signifying how deep a consensus for noise reduction we are exploring (Supplementary materials S3). Applying P_d_ to **‘AVIT**, we retained 16/15/15 (normal/strong/extra-strong) members in *WST*. Similarly, we retained 21/19/16 (normal/strong/extra-strong) members in *GFS*, 4/4/2 (normal/strong/extra-strong) members in *MIS* and 1/1/1 (normal/strong/extra-strong) member in *ECS* (Fig. 3D). Comparisons of the correctly/incorrectly retained/rejected members using P_6_ parameters are provided in Supplementary materials. Knowing how the starting 1 member (i.e. *E. coli*) should match up to 1 distinct taxa (Fig. 3D), we compared how **‘AVIT** performed in each of the cases.

For *ECS*, irrespective of the method used, we filtered out noise and uniquely identified *E. coli* (Fig. 3D panel 1). In these samples 99.84–99.92% of sequences belonged to *E. coli* and average sequence depth was 734,280. Overall we had less noise and we could easily identify the main member. For *MIS*, taking all the 14 samples, 12.6-99.90% of the sequences belonged to *E. coli* with an average sequence depth of 126,050. While using *Rabc0.005* we retained 13 erroneous members, we retained 3 in normal and strong level and 1 in extra-strong level of **‘AVIT** (Fig. 3D panel 2). Using **‘AVIT**, we removed most of the potentially erroneous members. Analyzing the sequenced results we identified one sample as erroneous (Supplementary materials S4). After removal of this sample, irrespective of the method used, we filtered out all the noise and uniquely retained *E. coli* (Fig. 3D panel 3).

For *GFS*, we obtained an average sequence depth of 47,640 in 23 samples, with mean relative abundance of a member as 0.0043. While we expected no members, we retained 31 members using *Rabc0.005* and 21/19/16 (normal/strong/extra-strong) members using **‘AVIT**. Firstly, **‘AVIT** clearly lead to retaining lower number of *potentially* erroneous members. However, in all cases it was not *zero* (Supplementary Figure 8). In the absence of any evidence of contamination of the gnotobiotic isolator, and the low sequencing depth obtained from the GF samples, these sequences are likely amplified from the environment, reagents and/or derived from machinery.

Analyzing *WST*, where different subsets bring in different levels of noise, we retained 39 members using *Rabc0.005* instead of retaining only *E. coli* (Fig. 3D panel 4). The erroneous members retained reduced by >50% after using **‘AVIT**. Pooling all the samples together, study specific abundance and variance changed, so did the outcome of **‘AVIT**. This highlights the importance of pooling samples for analysis, consistent with our earlier observations in the mock community.

In the second part of the *in-vivo* studies, we collected fecal samples from Altered Schaedlers Flora (ASF) mice. Firstly, we took the raw sequences of the ASF flora^23^ and trimmed to the V4 region and matched the *in-silico* sequences to the RDP database. Accordingly, we obtained 7 distinct genera, *Parabacteroides*, *Mucispirillum*, *Lactobacillus*, *Clostridium XIVb*, *Dorea*, *Roseburia* and *Pseudoflavonifractor*, which represented the 10 members of ASF. Interestingly, after processing of the 13 fecal samples from the ASF mice, using same process as described earlier, we obtained 99 genus level taxa. Average sequence depth was 449,765 (Fig. 3B). Removal of singletons had no effect on noise reduction (Fig. 3C). Using *Rabc0.005* or using **‘AVIT** at any stringency level using P_d_ parameters we retained only *Parabacteroides* and *Lactobacillus* (Fig. 3D panel 5). Members retained/rejected using the P_6_ parameter sets are provided in Supplementary data.

Overall, using the *in-vitro* and *in-vivo* studies; a) we identified parameter sets P_6_ and P_d_ for **‘AVIT**, enabling us to infer true members in highly or poorly diverse microbial communities, respectively, b) **‘AVIT** performed equal if not better than *Rabc0.005* in terms of retaining less erroneous members, more so with erroneous samples, and c) pooling of samples into proper bins facilitated identification of true/erroneous members.

### ‘AVIT and categorization of human gut microbiome

**Figure 4:**
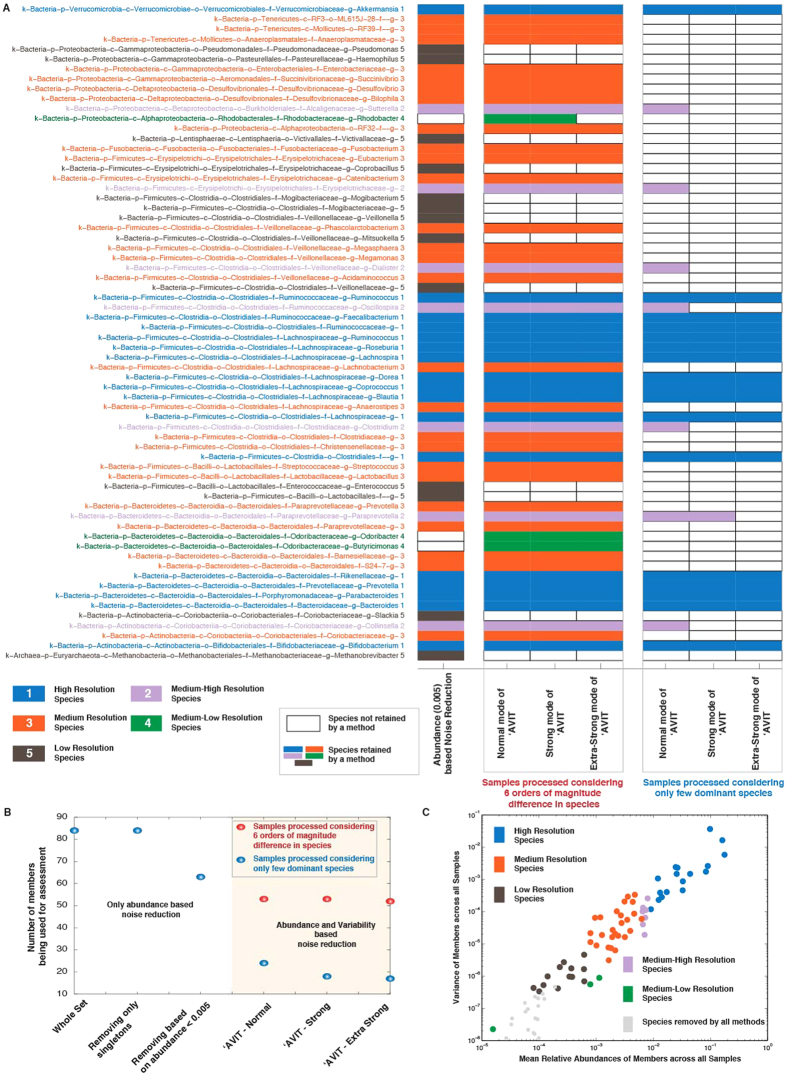
Application of ‘***AVIT*** for fecal samples from Lauber *et al.*^16^ including samples from lean healthy, obese diabetic and obese non-diabetic individuals before and after fiber supplementation. As compared to abundance only method of noise reduction, using ‘***AVIT*** we firstly retain a different set of members. Additionally, based on parameter choices and stringency levels in ‘***AVIT***, we retain different sub-groups of members. Based on the presence/absence of members across different methodologies, we categorized the members in terms of high to low resolution. Blue: high resolution – Category 1, Purple: medium-high resolution – Category 2, Orange: medium resolution – Category 3, Green: medium-low resolution – Category 4 and Brown: low resolution – Category 5. This categorization of members allowed us to implicitly consider this scoring downstream for analysis and for rank ordering hypothesis generation.

Having benchmarked and parameterized **‘AVIT** in both *in-vitro* and *in-vivo* studies, we used **‘AVIT** to analyze sequencing data from clinical samples^16^. For all the 159 fecal samples, DNA extraction was performed and subsequently sequenced using one MiSeq machine (details in Lauber *et al.*^16^), and we obtained 84 genus level taxa (Fig. 4).

Removal of singletons had no effect on potential noise reduction (Fig. 4B). Using *Rabc0.005*, we retained 63 members (Fig. 4A). Subsequently we applied **‘AVIT** at different stringency levels (normal, strong and extra-strong) using both P_6_ and P_d_ parameters to investigate a spread of potential noise reduction and accordingly a wide spectrum of potential membership of the microbial community.

Applying **‘AVIT** using P_6_ parameters, we retained 53/53/52 (normal/strong/extra-strong) members. In comparison, using P_d_ parameters, we retained 24/18/17 (normal/strong/extra-strong) members (Fig. 4, tabulated in Supplementary materials). Based on the retention/rejection of members across different **‘AVIT** implementations (combinations of different parameter sets and stringency levels), we categorized the microbial members into five bins (Fig. 4A). Consistent/sporadic retention/rejection of a member across different implementations could be a measure of *consistency*/*resolution*. High resolution bin (Fig. 4A) members were consistently retained across all implementations of **‘AVIT** and also retained using *Rabc0.005*. Medium-high resolution bin members were consistently retained across all implementations of **‘AVIT** using P_6_ parameter sets and retained for normal/strong level of **‘AVIT** using P_d_ parameter sets and retained using *Rabc0.005*. Medium resolution bin members were consistently retained using *Rabc0.005* and P_6_ but not P_d_ parameter sets across all implementations of **‘AVIT**. Medium-low resolution bin members were retained across some/all implementations of **‘AVIT** using P_6_ parameter sets but rejected using P_d_ parameter sets and *Rabc0.005*. Finally, low resolution bin members were consistently rejected across all implementations of **‘AVIT** using P_6_ and P_d_ parameter sets but retained using *Rabc0.005*. Projection of members in the average abundance versus variance across all samples is depicted in Fig. 4C. High resolution members are highly abundant and variable and consistently seen across all gut samples. *Akkermansia*, *Ruminococcus*, *Parabacteroides* and *Bacteroides* are some of the most commonly seen and implicated members of the gut community and are retained irrespective of the denoising method. On the contrary, low resolution members (e.g. *Veillonella*, *Pseudomonas*, *Mogibacterium*) with low abundance and low variance have relative abundance greater than 0.005 in a few samples but not seen consistently across samples. So while abundance based denoising would retain these members, **‘AVIT**, giving importance to variability rejects them. Medium-low resolution members were below the 0.005 relative abundance threshold but were variable enough to appear consistently across many samples. These members, including *Rhodobacter*, using abundance based cutoff would be rejected, but retained using **‘AVIT**. Interestingly, in the mock community studies *Rhodobacter* was identified as a difficult to detect genus (Fig. 2) but was retained according to the Medium-low resolution definition in the fecal samples. Thus taking into consideration how much and how often taxa appear we can assign a *consistency* and/or a *resolution* score for members to provide a ranking/weightage to aid in further downstream analysis using **‘AVIT**.

## Discussion

We present **‘AVIT**, an alternative approach to analyze metagenomic sequencing reads and provide insights into the application of 16S rRNA sequencing to elucidate microbial community composition. Our use of a defined *mock community* permits the calibration of parameters optimized to ensure best representation of communities under investigation. This is now standard practice within our laboratory and provides a reference permitting cross-comparison of different studies and samples run across different machines and different times.

In contrast to currently used abundance only methods for filtering, we present arguments about the potential issues, limitations and how they can be overcome by **‘AVIT**. One of the key outcomes of **‘AVIT** is to attain balance of *quality* and *quantity* for increased confidence in downstream processing. While using P_d_ parameters we focus on true dominant members, using P_6_ parameters, we explore potentially diverse microbial membership. **‘AVIT** is not a one-stop solution to completely de-noise a dataset. However, we demonstrated how coupling *in-vitro* mock communities and *in-vivo* defined studies we can use **‘AVIT** (with benchmarked parameters and different stringency levels) to explore a wide spectrum of potential membership of the microbial community in any clinical sample. Taking into account abundance, variability across samples and raw counts we ascertain a resolution measure, which can be subsequently used for further downstream analysis.

Our work also explores the limits of 16S rRNA sequencing to define the membership of a community. Detection and inclusion of different taxa were not rigorously consistent with abundance. One example, *Rhodobacter* was excluded using **‘AVIT** despite being among the most abundant species in the *mock community* (Fig. 2). This would suggest that there are challenges to detect this particular taxa using 16S rRNA-based methods. However, in the fecal sample analysis, *Rhodobacter* could be detected as a Medium-Low Resolution species only using the **‘AVIT** protocol (Fig. 4). This may reflect mis-classification of taxonomy, different *Rhodobacter* species being present in the fecal sample versus the *mock community*, differences in the environment of the *Rhodobacter* impacting on the DNA extraction, or other issues. These observations could also be interpreted as demonstrating an underappreciated representation of *Rhodobacter* within the gut microbiome. With efforts to move towards determining the functional potential of the microbiome from merely characterizing its members, **‘AVIT** may have a role to play in identifying ‘*low-abundant*’ taxa that play significant metabolic roles.

Our work also explores the limits of the utility of 16S rRNA sequencing to characterize microbial communities. The data summarized in Fig. 2 demonstrates a limited scope to reproduce known bacteria from mock communities of known composition. As demonstrated, **‘AVIT** significantly improves the reconstruction vs *ad hoc* methods, however, as the goals of studies move towards functional characterization, whole genome metagenomics might be beneficial. While currently demonstrated for 16S, **‘AVIT** framework is also applicable for similarly error-prone, noisy, community level ‘*omic*’ strategies.

## Additional Information

**How to cite this article**: Chakrabarti, A. *et al.* Resolving microbial membership using Abundance and Variability In Taxonomy (‘*AVIT*). *Sci. Rep.* **6**, 31655; doi: 10.1038/srep31655 (2016).

## Supplementary Material

Supplementary Information

Supplementary Part 1

Supplementary Part 2

Supplementary Part 3

Supplementary Dataset 1

Supplementary Dataset 2

Supplementary Dataset 3

Supplementary Dataset 4

Supplementary Dataset 5

Supplementary Dataset 6

Supplementary Dataset 7

Supplementary Dataset 8

Supplementary Dataset 9

Supplementary Dataset 10

Supplementary Dataset 11

Supplementary Dataset 12

Supplementary Dataset 13
