# Supplementary material for: Resolving microbial membership using Abundance and Variability In Taxonomy (‘AVIT )
Source: Sci Rep. 2016 Aug 17;6:31655. doi: 10.1038/srep31655 (PMC4987704; doi:10.1038/srep31655)
Supplement: Supplementary Information [file srep31655-s1.pdf]

Resolving microbial membership using Abundance and Variability In Taxonomy (*'AVIT*)

Anirikh Chakrabarti[1], Jay Siddharth[1], Christian L. Lauber[1], Mathieu Membrez[1], Bertrand Betrisey[1], Carole Loyer[1], Chieh Jason Chou[1], Zoltan Pataky[2], Alain Golay[2], Scott J. Parkinson[1*]

Supplementary Materials – Tables, Text and Figures

Supplementary Table S1: List of strains used for the mock community.

Accession ID	Name
DSM 20243	Lactobacillus gasseri
DSM 158	Rhodobacter sphaeroides
DSM 20478	Enterococcus faecalis
DSM 791	Clostridium beijerinckii
DSM 50071	Pseudomonas aeruginosa
DSM 20044	Staphylococcus epidermidis
DSM 20016	Lactobacillus reuteri
DSM 16379	Propionibacterium acnes
DSM 30007	Acinetobacter baumannii
DSM 20231	Staphylococcus aureus subsp. aureus
DSM 18039	Escherichia coli
DSM 2134	Streptococcus agalactiae
DSM 14662	Anaerostipes caccae
DSM 1447	Bacteroides vulgatus
DSM 13814	Dorea longicatena
DSM 22959	Akkermansia muciniphila
DSM 31	Bacillus cereus
DSM 19120	Actinomyces odontolyticus
DSM 19850	Blautia wexlerae
DSM 20088	Bifidobacterium longum subsp. infantis

S1 Mock Community and 'AVIT

Sequencing ribosomal genes from samples of an unknown taxonomic composition poses a conundrum. True biological variability of nucleotide sequences can be masked by PCR, PhiX concentration and sequencing errors of the target gene, which in turn could affect the apparent composition of the community. Knowing this, we began with a mock community of 20 defined bacterial taxa (tabulated in **Supplementary Table S1** and **Figure 2A**) and assessed whether **'AVIT** filtered inherent noise from the data, retaining

only the members of the mock community in the final dataset. For this part of the study, we formulated a mix of samples (referred further as *20Study1*), which included single strain samples (20 corresponding to one species in one sample), staggered pool samples (14) and equimolar samples (6), which were sequenced using one MiSeq machine and analyzed as one whole set. Depending on the database used to classify the sequences; we obtained between 132 and 148 genus level taxa using the MOTHUR MiSeq SOP (http://www.mothur.org/wiki/MiSeq_SOP) (details in supplementary data InVitro_40_Samples_Mix_RDP_Classified_Data.xls, InVitro_40_Samples_Mix_Silva_Classified_Data.xls, InVitro_40_Samples_Mix_GreenGenes_Classified_Data.xls). Within any level of noise reduction, we additionally have three parallel arms taking into account only the abundances within each sample (arm 1), both abundance and variability within each sample (arm 2) and both abundance and variability within the whole study (arm 3) to obtain different possible filtered states of the initial dataset (methodology illustrated in **Figure 1**).

Using **'AVIT** at different levels, we compared the amount of noise remaining in the dataset to currently used methods (i.e. abundance based) of noise reduction. For example, by removing only singletons (members identified in only one sample), we reduced around 7.2% of the noise in the RDP, 2.34% in SILVA, and 1.90% in GG classified datasets (**Supp Figure 1, panels A-C**).

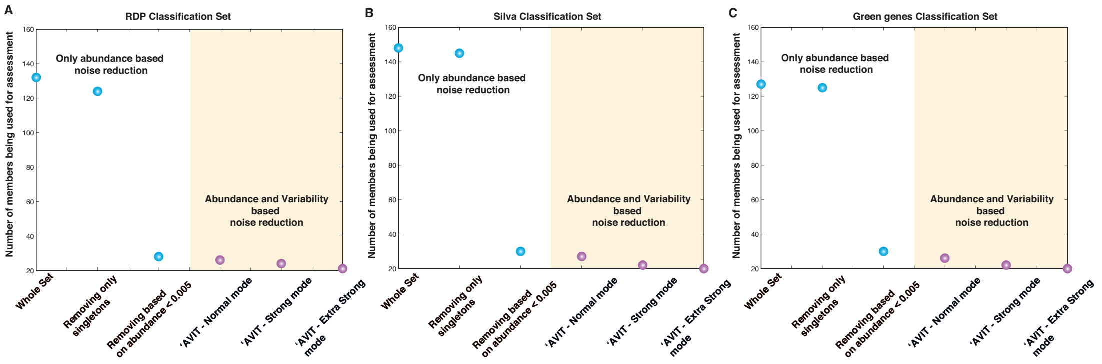

Supp Figure 1: Effect of different noise reduction methods on the final number of members used for downstream analysis for different taxonomical classification schemes (panel A - RDP, panel B - Silva and panel C - Greengenes) for *20Study1*. As opposed to abundance only based methods of noise reduction (blue circles), like removing singletons or hard abundance based cut-offs, incorporating both abundance and variability in noise reduction as proposed in **'AVIT** leads to improved noise reduction in the datasets (purple circles).

Using a strict relative abundance based cut off of 0.005, we reduced 92.8% of the noise in RDP, 92.18% of noise in SILVA, and 90.65% of noise in the GG classified datasets. In comparison, using both abundance and variability as proposed by **'AVIT** at different levels (normal, strong and extra-strong), we obtained ~93 % of noise reduction in normal level, ~95 % of noise reduction in strong level and ~97% in the extra-strong level consistently across the RDP, SILVA and GG classified datasets.

To demonstrate the specific effects and differences between the individual arms of **'AVIT**, we first look at the effect of the choice of proportionality

thresholds, ($\mathbf{P_{th}}$ = 0.0001, 0.0002… 0.9) alone in different arms while having other parameters fixed at $\mathbf{RC_{co}}$ = 1 and $\mathbf{CS_{co}}$ = 1 (**Supp Figure 2**).

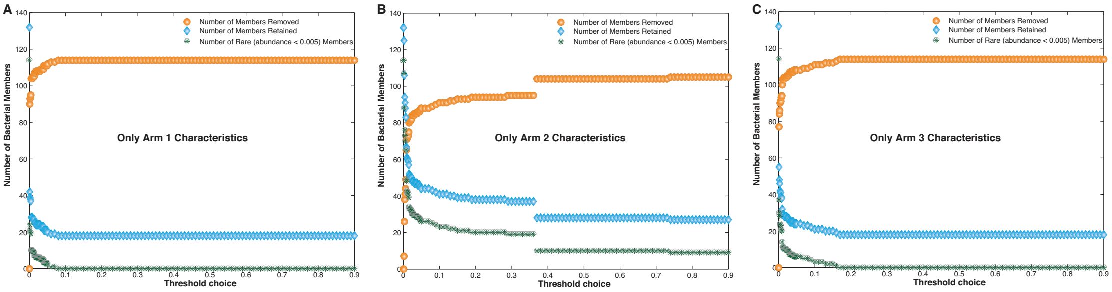

Supp Figure 2: Effect of choice of threshold in different arms (arm 1 – panel A, arm 2 – panel B and arm 3 – panel C) on number of members removed (orange circles), number of members retained (blue trapeziods) and number of rare members (defined as relative abundance < 0.005, green stars) for the RDP based taxonomical classification output of the *20Study1* study. Using arm 1 (as illustrated in Figure 1) by itself (panel A), by a threshold choice of ~0.06, we can remove all the noise and retain only the correct (known starting) members. Similarly for arm 3, by a threshold choice of ~0.16, we can remove all the noise and retain only the correct members. However for arm 2, even with a threshold choice of 0.9, we still have some noise remaining. Similar behavior is seen for the Silva and Greengenes based datasets (data not shown).

Since we knew the members in the mock community, we benchmarked how changing thresholds differentially allowed us to filter erroneously identified members from the dataset. We illustrate this by looking at the number of members removed, number of members retained and number of rare members (defined by relative abundance < 0.005). Using arm 1 by itself (removal of taxa based on maximum abundance within a sample alone) led to complete noise removal (i.e. the known starting 20 members remained after filtering), by a threshold choice of 0.06. If we increased the threshold any further, the number of retained members remained the same (**Supp Figure 2, panel A**). Similar observation was true for arm 3 as well (**Supp Figure 2, panel C**), which involved removal of taxa based on abundance and variability across the entire study (details in materials and methods), though at a higher threshold value of 0.16. However, when we used only the abundance and variability within a single sample (arm 2), a threshold value of 0.9 retained spurious taxa in the final output (**Supp Figure 2, panel B**). Consistent with removing all the noise, we removed all the rare members (defined as relative abundances < 0.005) across both arm 1 and 3. This was not the case for arm 2. Similar outcomes were observed with the SILVA and GG classified datasets (data not shown). Using just relative abundances within an individual sample while changing the proportionality threshold values ($\mathbf{P_{th}}$), it is possible to remove significant part of the noise in the dataset. Although a similar result was observed using abundance and variability in the whole dataset, at the individual sample level, abundance and variability did not result in removal of all noise.

S2 Stringency levels and retention of classified taxa

To demonstrate the core logic underlying **'AVIT**, we analyzed the results of mock community data processing after considering variability or abundance alone as well as increasing stringency levels of taxon removal. As shown in **Supp Figure 3**, abundance or variability alone is a poor predictor of noise as many taxa identified in the dataset were not present in the mock community.

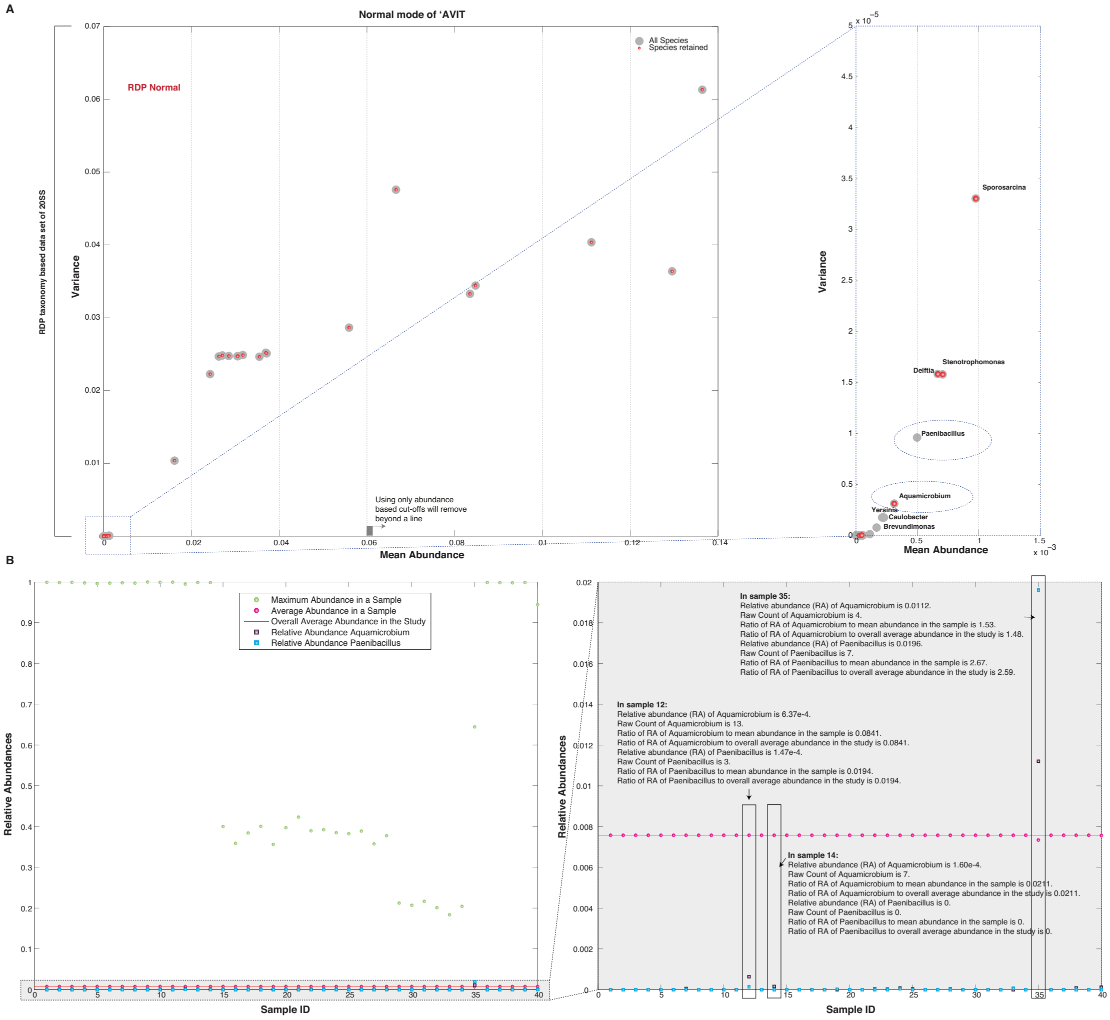

Supp Figure 3: (A) Variance versus mean abundance plot for the different members of the RDP classified dataset of *20Study1*. Grey filled circles indicate all the members, while red circles indicate members which are retained after normal level noise reduction. Top right panel indicates a zoom into the region with mean abundance less than 0.0015 and variance less than 0.00005. (B) Key decision making elements in filtering of members in the study. Green circles indicate maximum abundance within a sample, pink circles indicate average abundance within a sample, red line indicates average abundance of the whole study, purple squares indicate relative abundances of *Aquamicrobium* and blue squares indicate relative abundances of *Paenibacillus*. Bottom right panel indicates a zoom into the lower part of the figure marked with relative abundance < 0.02. Sample 12, 14 and 35 are highlighted with key elements which impact the choice of *Paenibacillus* as noise.

Had abundance been considered as the only metric, taxa on the right side of the hypothetical vertical line marking the abundance-based cut-off would have been retained. Similarly, had variance been considered as the only metric, taxa above the hypothetical horizontal line (marking the variance-based cut-off), would have been retained. However using the consensus based approach in **‘AVIT** at normal level of stringency we were able to retain known members while removing taxa that were not part of the mock community. As an example, *Paenibacillus* and *Aquamicrobium* were not part of the mock community but were selectively retained depending on the stringency of **‘AVIT.** In order to retain members following classification, the first step in each arm requires that the relative abundance of the member must be greater than a threshold ($\mathbf{P_{th}}$) times the maximum abundance in a sample (arm 1), average abundance in a sample (arm 2) and average abundance of the study (arm 3). Though overall mean abundance and variance of *Paenibacillus* was higher than *Aquamicrobium*, sequential application of the different arms and sub-steps in **‘AVIT** resulted in inclusion of *Aquamicrobium* and exclusion of *Paenibacillus*. Focussing on the $\mathbf{P_{th}}$ criterion, in all but four and two samples respectively*, Aquamicrobium* and *Paenibacillus* entries were excluded as they were consistently within the $\mathbf{P_{th}}$ range ($\mathbf{P_{th}}$ = 0.0001, 0.0002… 0.01) across all three arms (**Supp Figure 3B**). Subsequently, in the second sub-step of the arms (filtering based on $\mathbf{RC_{co}}$ corresponding to the normal level of **‘AVIT**), *Paenibacillus* was excluded since the raw count was in the range of $\mathbf{RC_{co}}$, leaving *Paenibacillus* exclusively in sample 35 (**Supp Figure 3B**). Subsequently, when we applied the third sub-step of the arms of **‘AVIT** (filtering based on $\mathbf{CS_{co}}$), *Paenibacillus* was excluded globally as it was present in only one sample. If we did not have the $\mathbf{CS_{co}}$ based filtering step now, *Paenibacillus* would never be removed. This highlights the specific importance of $\mathbf{CS_{co}}$ step. Considering the case of *Aquamicrobium,* the second sub-step of filtering (based on $\mathbf{RC_{co}}$**)** led to removal of the entry for sample 35 alone due to the raw count. This left three other instances of *Aquamicrobium* containing samples. Subsequently, when we filtered using $\mathbf{CS_{co}}$ in the third sub-step, *Aquamicrobium* was present in 3 samples (**Supp Figure 3B**), therefore it was not filtered out. Thus, using a combination of raw counts, relative abundances within and across specific samples, and presence across the study, we were able to incorporate both variability and abundance to obtain a more refined dataset. Nevertheless, at the normal level of **‘AVIT**, we were not able to remove all the erroneous members. This is where the different stringency levels come into play (**Figure 1B**, details in materials and methods).

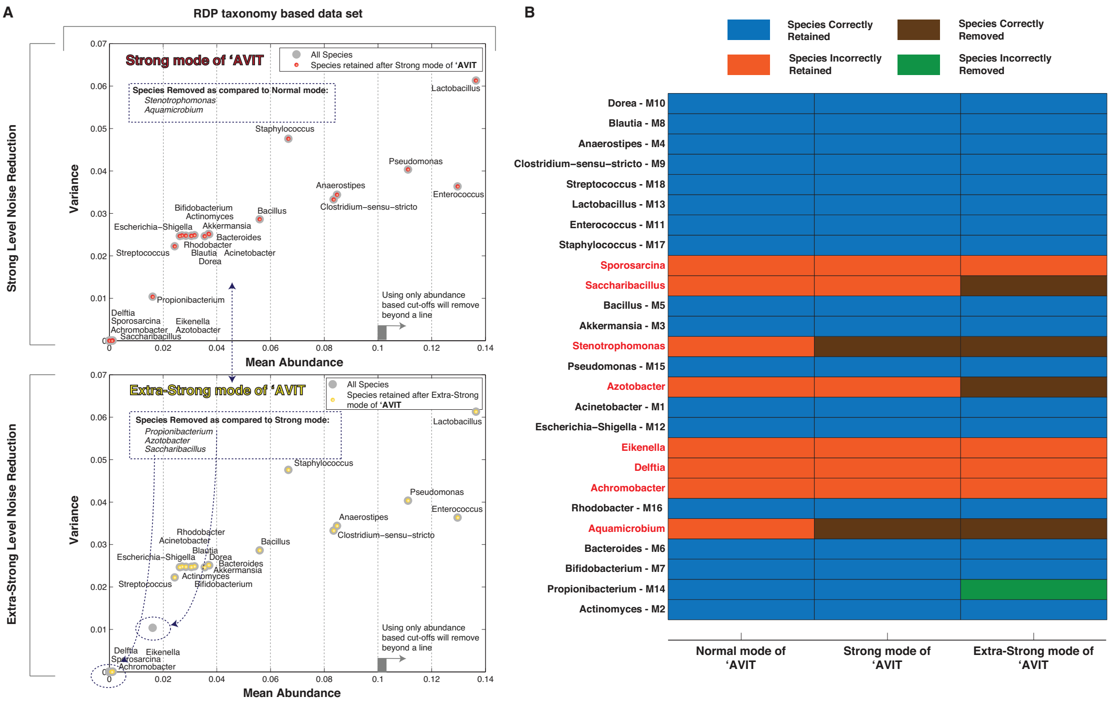

Supp Figure 4: (A) Variance versus mean abundance comparison for different species identified using the RDP classification for *20Study1*. Similar plot showing the characteristics for members identified using Silva classification and Greengenes (GG) classification can be seen in *Supp Figure 5*. Top plot of panel A corresponds to strong mode of **'AVIT** while bottom plot of panel A corresponds to extra-strong mode of **'AVIT**. Grey circles indicate all the members, while red circles (in top plot) and yellow circles (in bottom plot) indicate members which are retained after noise reduction using different modes of **'AVIT**. Arrows and dotted boxes indicate the specific species removed in one as compared to the other. (B) Heatmap representing retention and rejection of species across different modes of **'AVIT**. Blue and brown filled boxes indicate species correctly retained or removed after application of any mode of **'AVIT**, while red and green filled boxes indicate species that were incorrectly retained or removed.

In the RDP dataset (**Supp Figures 3 and 4**), as we increased the stringency level from normal to strong, two additional species were removed; *Stenotrophomonas* and *Aquamicrobium.* As explained earlier, *Aquamicrobium* was not removed using the normal level of stringency. However, using the strong level of **'AVIT**, two of the three samples retained using the normal level (in the range of $\mathbf{RC_{co}}$ = 5 to 8), were removed. Thus, the remaining entry after the second sub-step was subsequently filtered in the third, based on the $\mathbf{CS_{co}}$ parameter ($\mathbf{CS_{co}}$ = 1). Similarly, *Stenotrophomonas* was found in 24/40 samples, all having counts ≤ 8, except for sample 35 with a count of 9. *Stenotrophomonas* is a common contaminant of plasticware and therefore could be observed due to contamination of tubing, aerosols or from misclassification. In the normal level of **'AVIT**, 5 samples containing *Stenotrophomonas* remain with raw counts ranging from 5-9 per sample. However, using the strong level, one sample remains which is subsequently removed based on the $\mathbf{CS_{co}}$ parameter ($\mathbf{CS_{co}}$ = 1).

In the extra-strong level of **'AVIT,** we additionally removed *Propionibacterium*, *Azotobacter* and *Saccharibacillus* (**Supp Figure 4, top to bottom panel**). While removal of *Azotobacter* and *Saccharibacillus* was correct, removal of *Propionibacterium* was not. *Saccharibacillus*, was found in 12/40 samples, all having counts ≤ 9 (with the exception of sample 7 with a count of 38). Similarly, *Propionibacterium* was found in 12/40 samples, all having counts ≤ 9 (with the exception of sample 35 with a count of 230). So at extra-strong level, we removed all instances with individual counts ≤ 10. Thus, similar to *Saccharibacillus,* one sample containing *Propionibacterium* remained that was subsequently removed in the third sub-step despite the fact that *Propionibacterium* formed part of the mock community. Interestingly, this particular sample (number 35) exclusively contained *Propionibacterium.* The relative difficulty of retaining *Propionibacterium* may be related to inherent challenges of 16S amplification for this species due to primer biases. Thus, deploying stricter levels of **'AVIT**, certain taxa may mistakenly be treated as noise and removed from downstream analysis. In our case, the sequence depths varied from 357 reads (for single barcode sample 35 containing *Propionibacterium sps),* to 97,683 reads with a mean of 46,962 reads across all samples. Spiking amplicon based sequencing runs with a known strain mix is good wet lab QC practice. Routine use of a mock community in combination with **'AVIT** could help to discern genuine membership within complex biological samples.

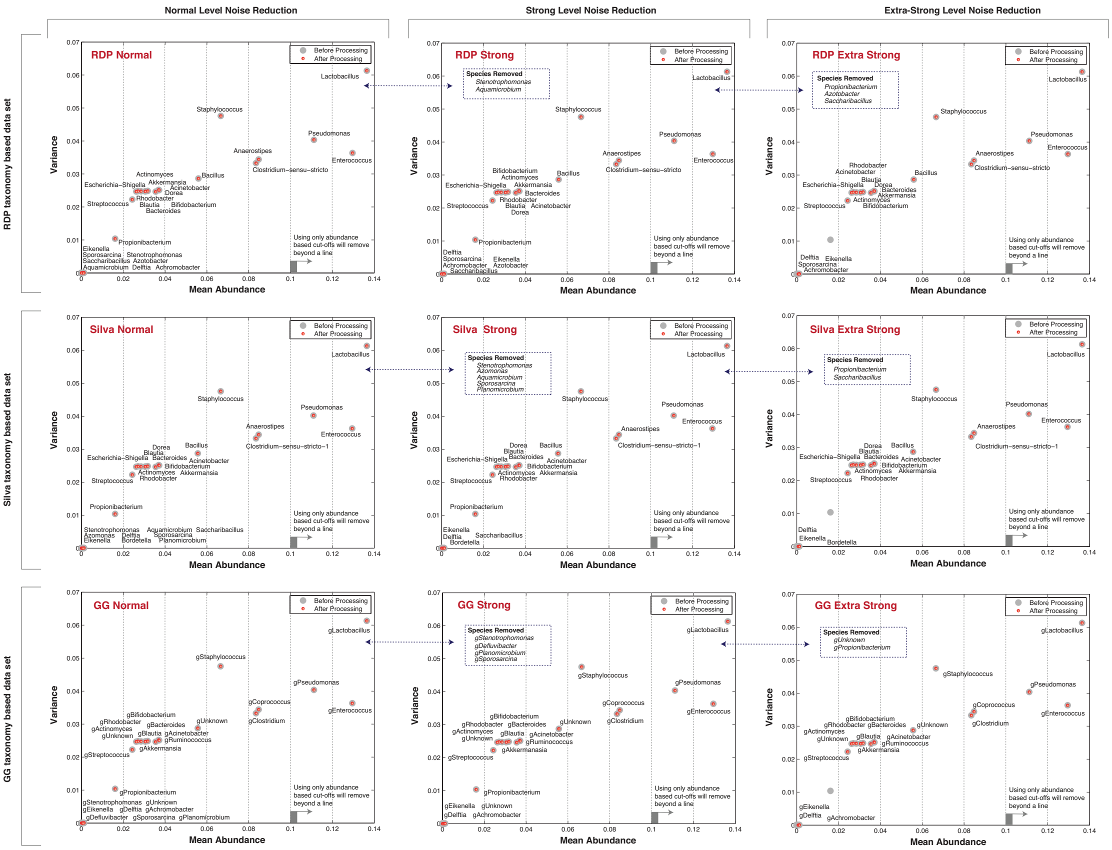

Supp Figure 5: Variance versus mean abundance comparison for identified members using different classifications for *20Study1*. First row corresponds to members identified using the RDP classification, middle row for Silva classification and bottom row for Greengenes (GG) classification. First column corresponds to normal noise reduction mode of **‘AVIT**, middle column for strong noise reduction mode of **‘AVIT** and third column for extra-strong noise reduction mode of **‘AVIT**. Grey filled circles indicate all the members, while red circles indicate members which are retained after noise reduction at each step. Arrows and dotted boxes inside second and third column plots indicate the specific species removed in one as compared to the other. For example, in strong noise reduction scheme for RDP (top row) as compared to normal noise reduction, we have additionally removed *Stenotrophomonas* and *Aquamicrobium*.

S3 Parameterization of ‘AVIT

Output of **‘AVIT** is dependent on the parameters used. Accordingly, two sets of parameters were identified;

a) from the *in-vitro* experiments (P_6) to optimize retention of true members in a six-orders of magnitude variable microbial community,
b) from the *in-vivo* experiments (P_d) to optimize retention of true members for a community dominated by only few dominant members.

P_6 is characterized as – $\mathbf{P_{th}}$ = 0.0001, 0.0002, $\mathbf{RC_{co}}$ = 1, 2… 10 and $\mathbf{CS_{co}}$ = 1 while P_d is characterized as – $\mathbf{P_{th}}$ = 0.0001, 0.0002… 0.01, $\mathbf{RC_{co}}$ = 1, 2… 50 and $\mathbf{CS_{co}}$ = 1.

Furthermore, we investigated the $\mathbf{RC_{co}}$ parameter choice, which is the determining factor for different stringency levels i.e. normal, strong and extra-strong. Having accounted for the presence/absence of a member in arm 2 and arm 3 of **'AVIT,** we investigated the impact of replicate and raw count numbers used for cutoffs in **'AVIT** in the cases of *EMS*, *P1S* and *P2S*. We simulated combinations of $\mathbf{RC_{co}}$ (1 to 500), the number of replicates used for analysis and analyzed the number of taxa retained for normal level of **'AVIT** (**Supp Figure 6**). The number of replicates (using $\mathbf{CS_{co}}$ parameter = 1) had a two-way impact on noise reduction. As the number of replicates moves from smaller to larger numbers, the noise reduction decreases in stringency. Using any given $\mathbf{RC_{co}}$ cutoff for *P1S* and *P2S*, 3-7 replicates provided the most robust outcomes from **'AVIT**. In terms of $\mathbf{RC_{co}}$, as we increased $\mathbf{RC_{co}}$ from 1 to 500, there was a continuous decrease in the number of members retained. In the cases (i.e. *EMS*, *P1S* and *P2S*) we analyzed, increasing $\mathbf{RC_{co}}$ had no impact beyond value of 9. This observation was consistent and forms the rationale for our choice of $\mathbf{RC_{co}}$ values in the range of 1-4 for normal level of **'AVIT**, 5-8 for strong level and 9-10 for extra-strong level. $\mathbf{RC_{co}}$ values in the range of 1-10 balance the retention or removal of less abundant, potentially valid community members.

S4 *In vivo* erroneous sample identification

Considering all the samples in the *in vivo* study, sequence depth ranged from 246 to 2,579,441. The sample (part of the monoinoculated mice fecal samples) with only 246 as sequence depth was considered as an erroneous sample. After removal of this sample, the sequence depth ranged from 4,979 to 2,579,441 with an average sequence depth of 155,650 (details in supplementary data MIGF_Supp_Data_AVIT.xls).

S5 *Member retention/rejection using 'AVIT with P_d parameter set for mock community in-vitro study*

Details of using P_d parameter sets including members retained and rejected in supplementary data InVitro_Equimolar_Samples_Data_AVIT.xls, InVitro_Pool1_Data_AVIT.xls, InVitro_Pool2_Data_AVIT.xls and InVitro_SingleStrain_Samples_Data_AVIT.xls. Additionally, comparison of the retained/rejected members across *EMS*, *P1S*, *P2S* and *SSS* samples are shown in **Supp Figure 7**. Clearly, using P_d parameter sets, we retained absolutely no noise in any of the samples in the *in-vitro* studies. But since our objective was to optimize retention of more true members, member retention using P_6 parameter set is shown in main **Figure 2**.

S6 *Member retention/rejection using 'AVIT with P_6 parameter set for in-vivo studies*

Details of using P_6 parameter sets including members retained and rejected in supplementary data in MIGF_Supp_Data_AVIT.xls for Monoinoculation study and ASF_Supp_AVIT_Data.xls for ASF study.

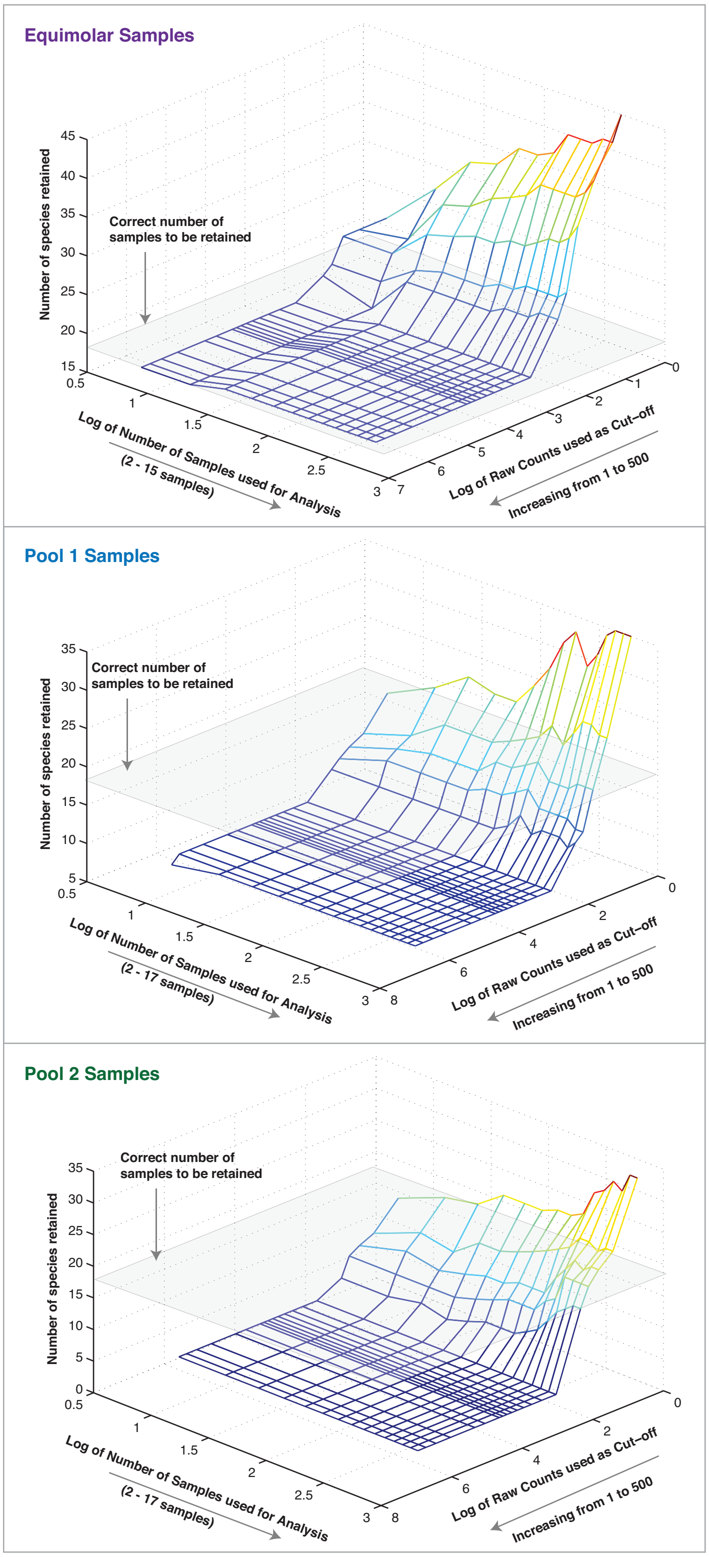

Supp Figure 6: Output of **'AVIT** (in terms of number of species retained) for different combinations of number of samples used for analysis (ranging from 2 – 15 in case of Equimolar samples and ranging from 2 - 17 in case of Pool 1 and Pool 2 samples) and different raw counts used for cut-off (ranging from 1 – 500).

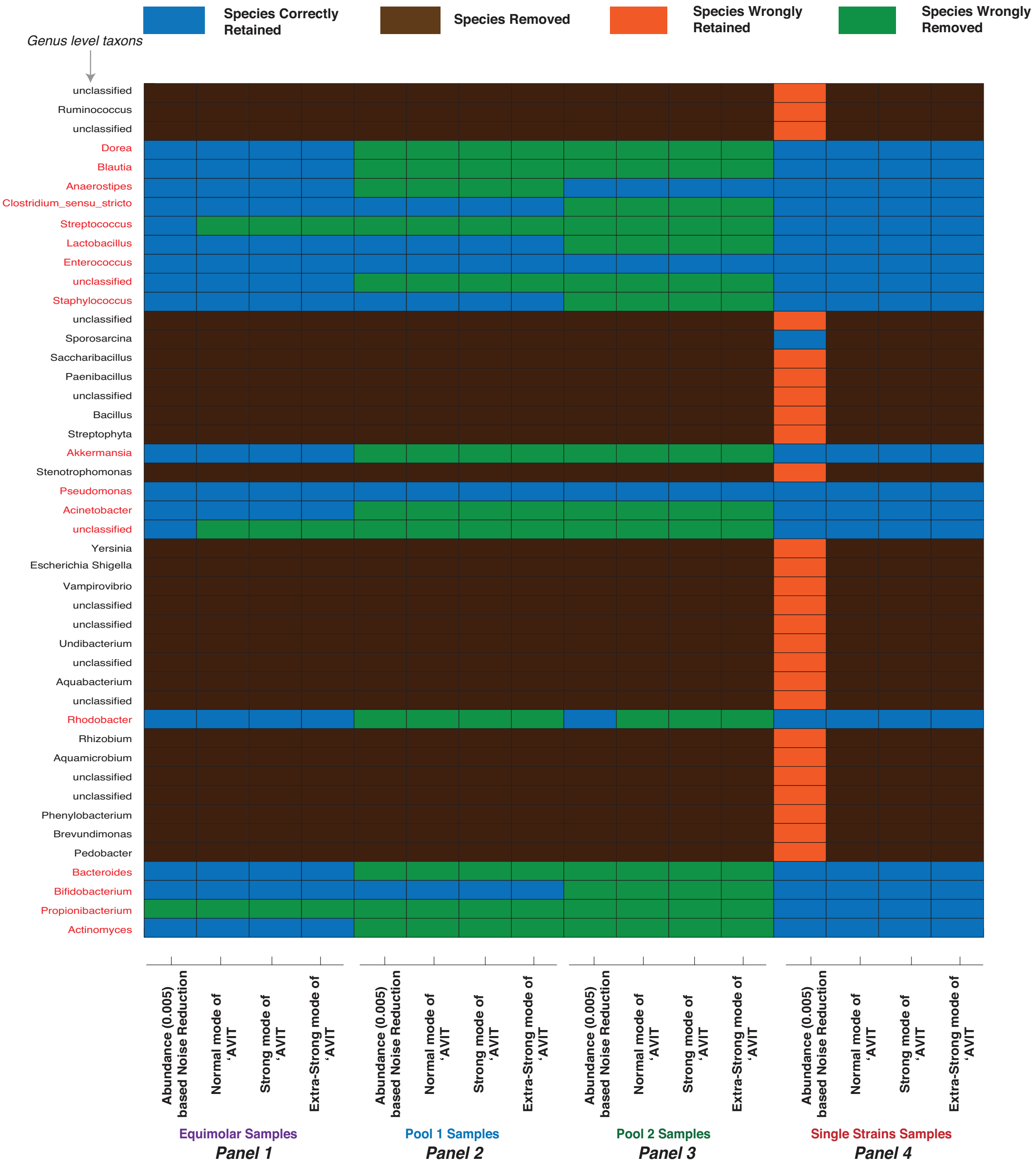

Supp Figure 7: Member retention/rejection using **'AVIT** with P_d parameter set for mock community *in-vitro* study.

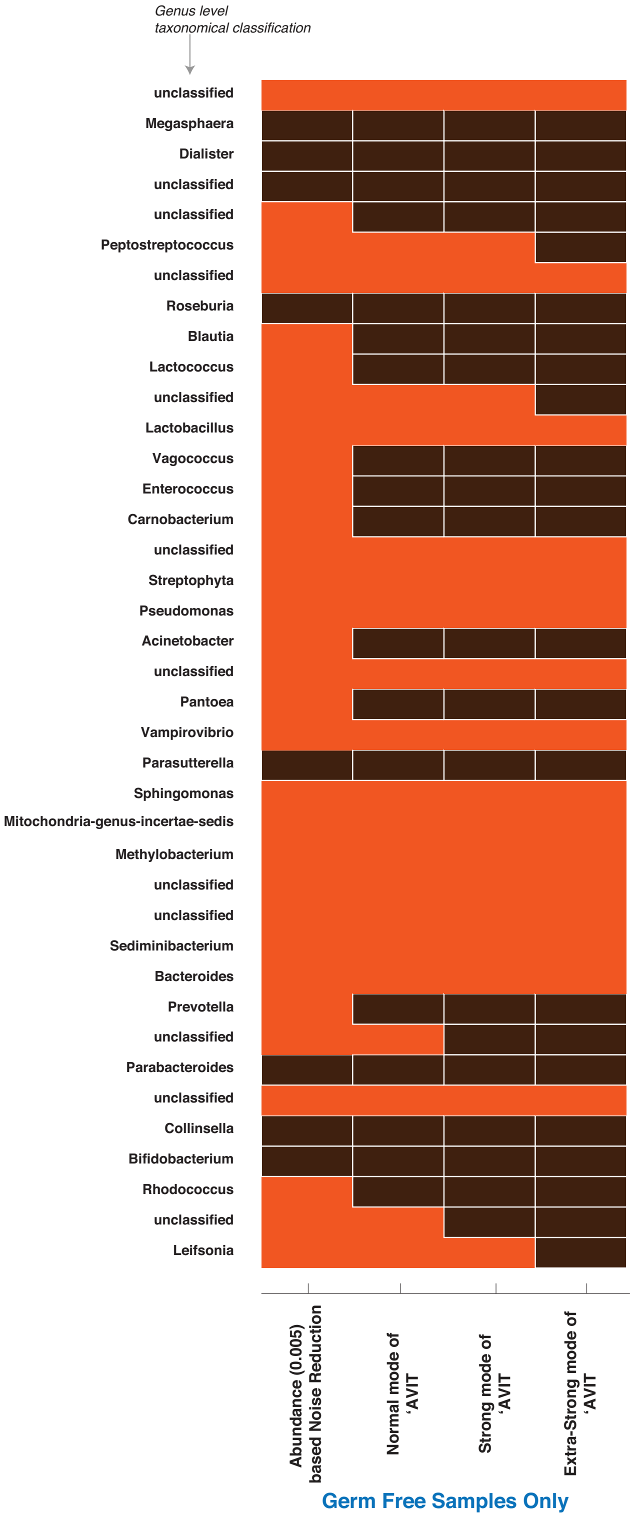

Supp Figure 8: Member retention/rejection using **'AVIT** with P_d parameter set for Germ-free (GF) samples only. Red filled boxes indicate members retained and brown filled boxes indicate members rejected.

S7 *Using 'AVIT in Octave*

'AVIT has been tested and can be used on open source GNU Octave. Last check was done on version 3.8.2. In case of running in Octave, two flags have to be modified as a) Do_you_want_checks should be 0 and b)

Do_You_Want_Plot should be 0. For reference, please see the sample file provided as Analyze_MockCommunity_Study_1_Data_Using_AVIT.m.
